# Comparative Weight Change With Initiation and Adherence to Common Medications for Type 2 Diabetes

**DOI:** 10.1002/oby.70022

**Published:** 2025-10-16

**Authors:** Nadia M. Sneed, Sheryl L. Rifas‐Shiman, Joshua Petimar, Jessica G. Young, L. Charles Bailey, Matthew F. Daley, David M. Janicke, W. Schuyler Jones, Carly Prentice, Jason P. Block, William J. Heerman

**Affiliations:** ^1^ School of Nursing Vanderbilt University Nashville Tennessee USA; ^2^ Department of Population Medicine Harvard Medical School and Harvard Pilgrim Health Care Institute Boston Massachusetts USA; ^3^ Department of Epidemiology, Harvard T.H. Chan School of Public Health Boston Massachusetts USA; ^4^ Institute for Health Research, Kaiser Permanente Colorado Aurora Colorado USA; ^5^ Department of Clinical and Health Psychology College of Public Health and Health Professions, University of Florida Gainesville Florida USA; ^6^ Department of Medicine Duke University Health System Durham North Carolina USA; ^7^ Faith Family Medical Center Nashville Tennessee USA; ^8^ Department of Pediatrics Vanderbilt University Medical Center Nashville Tennessee USA

**Keywords:** glucagon‐like peptide‐1 receptor agonists, obesity, sodium‐glucose cotransporter 2, target trial, type 2 diabetes

## Abstract

**Objective:**

This study aimed to estimate population‐level effects on weight change of initiating/adhering to additional glucose‐lowering medications in adults with type 2 diabetes prescribed metformin.

**Methods:**

We conducted a target trial using electronic health record data from 22,601 patients (age 20 to < 80 years) prescribed metformin to determine initiation/adherence to dipeptidyl peptidase IV (DPP4) inhibitors, glucagon‐like peptide‐1 receptor agonists (GLP‐1RAs), sodium‐glucose cotransporter 2 (SGLT‐2) inhibitors, long‐acting insulin, or sulfonylureas. Inverse probability weighting of marginal structural models with standardization by baseline covariates was used to estimate population‐level effects of initiating/adhering to different medications on average 24‐month weight change.

**Results:**

At 24 months, a mean −5.15 kg (95% CI −10.6, −1.36) and −6.71 kg (95% CI −8.38, −4.34) weight loss would be observed for initiation/adherence to GLP‐1RAs and SGLT‐2s respectively. At 6 months, weight loss for DPP4s would be observed (−0.89 kg, 95% CI −1.41, −0.32) though not at 12 or 24 months. Glimepiride would be associated with weight gain at 6 and 12 months (0.88 kg, 95% CI 0.44, 1.22; 1.01 kg, 95% CI 0.32, 1.51) but not at 24 months.

**Conclusions:**

Initiation/adherence to GLP‐1RAs and SGLT‐2s over 24 months could result in average weight losses of 5.15 kg and 6.71 kg, respectively.

**Study Importance**

- What is already known?
  ○ Findings from randomized clinical trial data suggest that glucagon‐like peptide‐1 receptor agonists (GLP‐1RAs) and sodium‐glucose cotransporter 2 (SGLT‐2) inhibitor drug classes result in relatively large reductions in body weight, between 3.0 and 5.7 kg in total over ~6–12 months.
- What does this study add?
  ○ We conducted a pragmatic comparison of the effects between glucose‐lowering medication classes on weight change to determine which medication classes may have the most favorable effects on weight in clinical settings.
  ○ In adults with type 2 diabetes (T2D), we found that treatment with metformin plus GLP‐1RAs or SGLT‐2s could result in an average absolute weight loss of 5.15 kg or 6.71 kg, respectively, over 24 months.
- How might these results change the direction of research or the focus of clinical practice?
  ○ These findings highlight differences in expected weight change after prescribing medications for adults with T2D.

## 1 Introduction

For adults living with type 2 diabetes (T2D), the goals of treatment are to achieve remission and support body weight reduction, if possible [1, 2], and prevent comorbid complications that include both macrovascular and microvascular disease [3]. Glucose‐lowering medications in combination with lifestyle therapies (e.g., intensive lifestyle modification programs, including dietary modification and physical activity) are recommended as first‐line treatment for the almost ~37 million United States (U.S.) adults living with T2D [4, 5]. There are currently 10 classes of oral and injectable glucose‐lowering drug classes available to treat T2D, with each having varying effects on body weight. Metformin, often prescribed at the onset of T2D as a monotherapy, is considered weight neutral or to be associated with mild weight loss [5]. However, other drug classes, typically added in combination with metformin, including sulfonylureas and insulin, have been shown to cause weight gain [5]. Newer medications—sodium‐glucose cotransporter 2 (SGLT‐2) receptor inhibitors and glucagon‐like peptide‐1 receptor agonists (GLP‐1RAs)—alternatively promote weight loss [6, 7, 8].

Weight loss is a critical component of T2D treatment and management [5], particularly since excess body weight results in greater insulin resistance and worsened glycemic control [9]. Promising findings from randomized clinical trials (RCTs) suggest that SGLT‐2 and GLP‐1RA drug classes result in relatively large reductions in body weight, between 3.0 and 5.7 kg in total over ~6–12 months, respectively [7, 8]. However, there is limited comparative data on long‐term weight effects from patients receiving glucose‐lowering medications. Furthermore, estimated effects of medications on weight loss come from RCTs where the control group receives a placebo rather than other glucose‐lowering medications [10, 11]. A systematic review and meta‐analysis of 25 RCTs reported a mean weight reduction of 2.8 kg in adults with T2D taking high‐dose GLP‐1RAs. Yet similar weight loss effects were also observed in controls taking other glucose‐lowering medications that have been previously associated with weight gain or no weight change [12]. While there is less trial data on weight loss outcomes for SGLT‐2 inhibitors, studies have reported comparable weight loss outcomes to those of studies conducted with GLP‐1RAs [6, 13]. Given the limitations and significant heterogeneity between study findings for SGLT‐2 and GLP‐1RA drug classes, especially in comparison to other glucose‐lowering medications, a pragmatic comparison of the effects between all glucose‐lowering medications on weight gain/loss is needed to establish which medication classes may have the greatest benefit on weight [14]. Because metformin has traditionally been used as a first‐line treatment for T2D [5], studies can only reasonably support comparisons of additional medications used after treatment with metformin.

Using electronic health record (EHR) data from eight health systems, we conducted a large cohort analysis to compare weight change across the most commonly prescribed classes of glucose‐lowering medications among patients who had a previously documented metformin prescription. The objective was to estimate the population‐level average causal effect of initiating and adhering to multiple second‐line treatments in individuals with T2D who had been prescribed metformin.

## 2 Methods

We obtained EHR data from eight health systems that were current or former participants in PCORnet, the National Patient‐Centered Clinical Research Network [15, 16], and identified patients who were incident users of common glucose‐lowering medications after previously having documentation of a prescription for metformin (Table S1). The Institutional Review Board (IRB) of Harvard Pilgrim Health Care approved this study as a single IRB for each of the healthcare systems contributing data.

The analytical approach used for this study can be understood as an application of “target trial emulation” [17]. This approach uses the protocol of a hypothetical randomized trial by applying strict inclusion and exclusion criteria and clearly defined study outcomes (“emulation” of the target trial) [18]. Table S2 summarizes a target trial and details of its emulation for our application and describes how each of the decisions related to this target trial emulation overlapped with a hypothetical RCT. In addition, statistical methods are used to account for the lack of random treatment assignment (e.g., inverse probability weighting) [19]. Importantly, these decisions were made prior to conducting the statistical analyses, similar to the approach taken in a clinical trial. Details regarding these methods using EHR data have been previously published [18]. One of the strengths of this approach is that it allows for exploration of initiation of medications (similar to an intent‐to‐treat approach from a trial) and adherence to medications over time (similar to a per‐protocol approach) [19]. In this study, the adherence models were our primary analysis because we wanted to examine actual uses of medications with weight outcomes; initiation‐only models were secondary.

### 2.1 Eligibility for Study Inclusion

**FIGURE 1:**
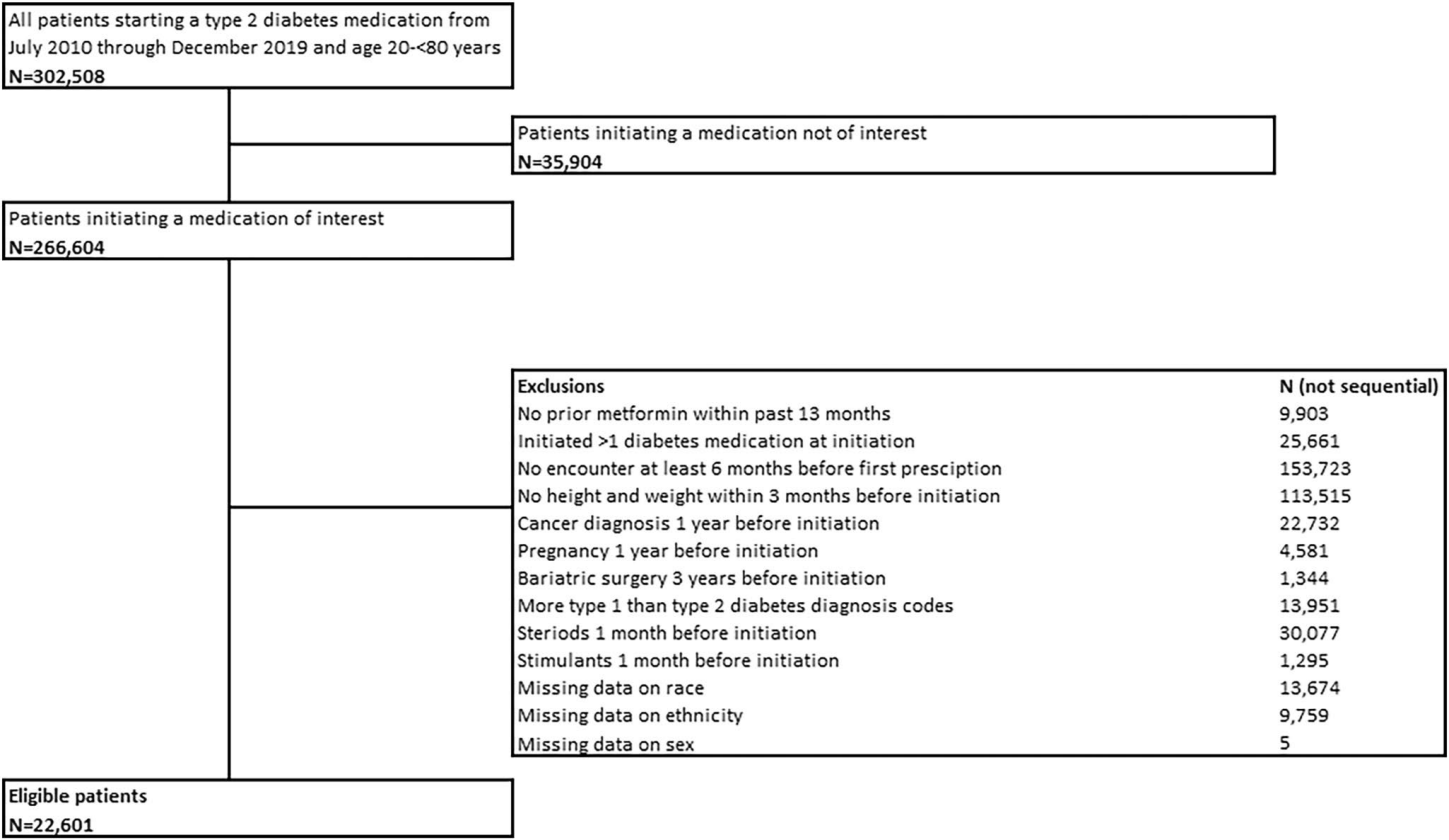
Flow diagram of eligible patients included in analysis.

Patients were included for analysis if they met the following eligibility (inclusion/exclusion) criteria: (1) healthcare system encounter between July 1, 2010, and December 31, 2019 (with baseline the time point a patient would first meet all eligibility criteria); (2) age 20 to < 80 years at baseline; (3) prior metformin prescription within 13 months of initiation of another medication for treatment of T2D; (4) no previous history of additional glucose‐lowering medications other than metformin to examine new initiation of medications in this class; (5) prescription for only one of the following glucose‐lowering medications prescribed in addition to prior metformin: dipeptidyl peptidase IV (DPP4s), GLP‐1RAs, SGLT‐2s, long‐acting insulin, glimepiride, and glipizide with no prior prescriptions for any of these medications prior to metformin use; (6) weight measure within 3 months prior to baseline to allow for calculation of the primary outcome of weight change; and (7) an encounter in the healthcare system at least 6 months prior to baseline (Figure 1).

We required prior metformin prescriptions because it was considered a first‐line therapy for almost all patients diagnosed with T2D during the study period and remains the most commonly prescribed first‐line treatment for T2D. We used 13 months as the time period for a documented prior metformin prescription to account for any metformin prescriptions in the year prior to initiation of additional diabetes medications. This allowed for a 1‐month grace period for refilling (considering that patients could have a 12‐month prescription, with a 90‐day supply and three refills).

Patients were required to have an encounter at least 6 months before the first glucose‐lowering prescription (other than metformin) to ensure that included patients were connected longitudinally to the healthcare systems from which their data were obtained. Confidence that patients were truly new users of one of the glucose‐lowering medications of interest would be lower if patients had < 6 months of history with the health system.

Similar to what would be expected in a clinical trial, the following exclusion criteria were included: diagnostic/procedure codes for cancer in the year prior, pregnancy in the year prior, bariatric surgery in the 3 years prior, and steroid or stimulant prescriptions within 1 month of the medication initiation date. These exclusions were necessary because of the substantial effects of each condition, procedure, and medication on weight. In addition, individuals were excluded if they did not have complete data on sex or race or ethnicity or if they had more documented diagnostic codes for type 1 diabetes than T2D.

### 2.2 Measures of Initiation and Adherence

The main exposures of interest were prescriptions for only one of the six oral or injectable glucose‐lowering medications. Glimepiride and glipizide were examined separately instead of by class (i.e., sulfonylurea) due to large numbers of eligible patients being prescribed these medications. All other medications were aggregated by subclass due to sample sizes being insufficient to examine individual medications.

To classify nonadherence to the initiated second‐line medication, we calculated the length of time patients were expected to have medications available, using information from the number of pills, days' supply, and number of refills in their prescriptions. The following assumptions were made for prescription information: If patients had missing refill information, we used only the number of pills or days' supply documented and assumed patients had no refills. We also assumed that prescriptions for ≥ 90 pills were for a 90‐day supply and that prescriptions for < 90 pills were for a 30‐day supply.

### 2.3 Outcomes

The outcome of interest was a change in weight (kg) measured at 6, 12, and 24 months after baseline (i.e., the difference between that measure and the baseline measure). Height and weight data were cleaned using the *growthcleanr* algorithm to remove implausible weight values [20, 21].

### 2.4 Covariates

The complete list of potential baseline and time‐varying adjustment covariates is reported in Table S3. Covariates included demographics, comorbidities, other prescription medications that could be associated with weight change, and further information about the timing of metformin prescriptions prior to the initiation of a new diabetes medication.

For all covariates and exposures, patients without a diagnosis, procedure code, or prescription were assumed to not have the disease, procedure, or prescription (e.g., if a patient did not have a recorded diagnosis of heart failure during baseline, it was assumed that the patient did not have the condition).

### 2.5 Statistical Analysis

Our analytic approach combines elements of previous work from the causal inference literature on marginal structural models [22, 23, 24, 25] to jointly accommodate the following various complicated features of this study [26]: (1) interest is in the effects of time‐varying treatment strategies that allow grace periods for time off treatment [27]; (2) appropriate adjustment for time‐varying confounders is needed; (3) missingness in the outcome of interest may be substantial relative to the baseline sample size at any selected post‐baseline follow‐up time such that some smoothing in outcomes over time is needed for adequate precision; and (4) baseline and time‐varying covariate adjustment to address selection bias due to informative outcome missingness is also needed.

We have previously applied this analytic approach in a comparative study of antidepressants and weight change where additional technical details are described [18]. In brief, this approach first calculates inverse probability weights (IPWs) that accomplish adjustment for potential time‐varying confounders/selection factors. Next a repeated outcomes model is fit to a person‐month dataset weighted by the IPWs. The dependent variable in this model is the outcome of weight change. The exposure was prescriptions for any of the six medications of interest (DPP4, GLP1‐RA, SGLT‐2, long‐acting insulin, glimepiride, glipizide); additional independent variables included a function of month (restricted cubic spline with four knots), treatment by month interaction terms, and potential baseline confounders/selection factors for missingness. The estimated model coefficients from the weighted outcome regression step are used to then “predict” the average weight change in that selected month under adherence to the treatment strategy defined by initiating that selected medication for all patients. Those predictions are then averaged to obtain a population‐level estimate of the average weight change in a selected month had all individuals in the eligible population adhered to that medication. We then estimated differences in population‐level estimates for each medication relative to the reference (GLP‐1RAs). We used a similar approach to estimate relative risks of gaining ≥ 5% of baseline weight compared to GLP‐1RAs. For this analysis, we only included data for the 6‐ and 12‐month time points due to a decline in the sample size at 24 months which resulted in wide and uninterpretable confidence interval (CI) estimates.

For our primary analyses, targeting effects of initiation and subsequent adherence, the IPWs were a product of two components: an “outcome measurement” IPW which depends on an estimate of the inverse probability of outcome measurement at each time, accounting for past measured time‐varying and baseline covariates, and an “adherence” IPW which depends on an estimate of the inverse probability of remaining adherent to the medication, also conditional on past measured time‐varying and baseline covariates.

Our secondary analyses, targeting effects of initiation only (regardless of adherence), were similar but did not involve an artificial censoring step; the IPWs only depend on the stabilized outcome measurement IPW. In all analyses, we truncated these IPWs at the 99th percentile [18]. We calculated 95% CIs by a nonparametric bootstrap of the entire procedure using 1000 bootstrap resamples. All analyses were conducted using SAS version 9.4 (Cary, NC).

Person‐time was censored upon evidence of death in all primary and secondary analyses. In a sensitivity analysis, we excluded deaths.

## 3 Results

After applying the eligibility criteria, *N =* 22,601 people met the eligibility criteria and were included at baseline in the analytic sample. Several differences in baseline demographic characteristics were evident across the subclasses of glucose‐lowering medications (Table 1). Notably, patients prescribed GLP‐1RAs were more likely to have obesity, to be identified as White race, and to have no documented diabetes recorded in their EHR; patients prescribed long‐acting insulin were more likely to be identified as Black race. Sulfonylureas were the most commonly prescribed medication subclass (26% glimepiride and 27% glipizide), followed by insulin (21%), DPP4s (12%), GLP‐1RAs (9%), and SGLT‐2s (5%). Among patients initiating GLP‐1RAs, 53.4% received liraglutide, 20.3% dulaglutide, 16.3% exenatide, 8.3% semaglutide, and 1.7% albiglutide. At baseline, the average BMI for the entire sample was 34.5 (SD 7.9) kg/m^2^, with 69% having obesity and 91% having a diagnosis code for T2D at baseline. There were a total of 400 deaths during the 24 months of follow‐up.

**TABLE 1:** Baseline characteristics (mean [SD] or %) of the study population.

						Sulfonylurea	
		DPP4	GLP‐1RA	Insulin	SGLT‐2	Glimepiride	Glipizide
	Overall	***n*** = 2667	***n*** = 2077	***n*** = 4794	***n*** = 1041	***n*** = 5911	***n*** = 6111
Characteristic	***n*** = 22,601	12%	9%	21%	5%	26%	27%
Age, years	56.0 (12.0)	58.0 (11.6)	51.3 (12.0)	54.7 (12.4)	54.7 (11.4)	57.7 (11.3)	56.5 (11.9)
Weight, kg	100.5 (25.4)	98.01 (24.2)	111.1 (27.2)	99.91 (26.5)	103.5 (24.5)	98.76 (24.1)	99.75 (24.8)
BMI, kg/m^2^	34.5 (7.9)	34.0 (7.7)	39.0 (8.4)	34.1 (8.5)	35.5 (7.6)	33.8 (7.3)	34.2 (7.6)
BMI category, %							
Normal	1704 (8)	218 (8)	20 (1)	518 (11)	43 (4)	433 (7)	472 (8)
Overweight	5277 (23)	662 (25)	232 (11)	1144 (24)	209 (20)	1556 (26)	1474 (24)
Obesity	15,620 (69)	1787 (67)	1825 (88)	3132 (65)	789 (76)	3922 (66)	4165 (68)
Sex = Female, %	11,113 (49)	1378 (52)	1436 (69)	2151 (45)	532 (51)	2754 (47)	2862 (47)
Race, %							
Asian	654 (3)	72 (3)	21 (1)	82 (2)	34 (3)	289 (5)	156 (3)
Black	5974 (26)	748 (28)	453 (22)	1695 (28)	239 (23)	1103 (19)	1736 (29)
White	14,413 (64)	1688 (63)	1526 (73)	2665 (56)	724 (70)	4087 (69)	3723 (61)
Other or > 1 race	1560 (7)	159 (6)	77 (4)	352 (7)	44 (4)	432 (7)	496 (8)
Ethnicity = Non‐Hispanic, %	20,532 (91)	2407 (90)	1870 (90)	4313 (90)	953 (92)	5441 (92)	5548 (91)
Type 2 diabetes, %	20,646 (91)	2353 (88)	1524 (73)	4455 (93)	865 (83)	5656 (96)	5793 (95)

Abbreviations: DPP4, dipeptidyl peptidase IV inhibitors; GLP‐1RA, glucagon‐like peptide‐1 receptor agonists; SGLT‐2, sodium‐glucose cotransporter 2 inhibitors.

### 3.1 Primary Outcome Analysis (Treatment Adherence Models)

**FIGURE 2:**
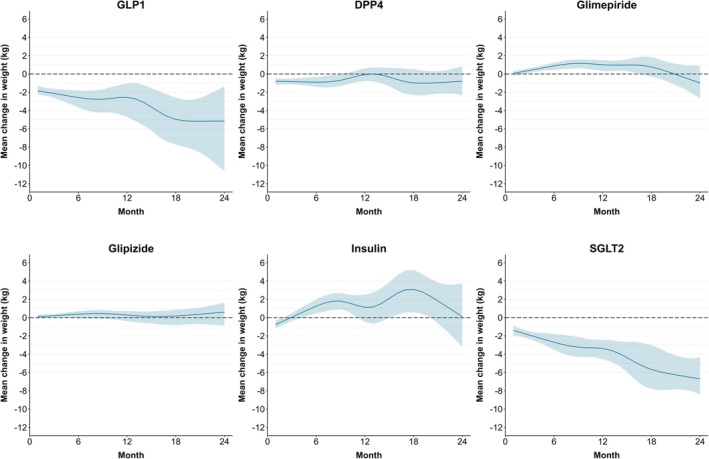
Associations of diabetes medications with weight change over 24 months. The figure shows the mean weight change (dark blue line) and 95% CIs from 1000 bootstrapped samples (light blue bands) for adherence to each of the six glucose‐lowering treatments over 24 months from initiation. The null (0 kg mean weight change) is depicted with a dashed horizontal line. DPP4, dipeptidyl peptidase IV inhibitors; GLP‐1RA, glucagon‐like peptide‐1 receptor agonists; SGLT‐2, sodium‐glucose cotransporter 2 inhibitors.

Compared to the reference, GLP‐1RAs, each of the subclasses of medications except SGLT‐2s showed greater weight change at each of the follow‐up time points (Table 2). At 24 months, we estimated that initiation and adherence to DPP4s, long‐acting insulin, glimepiride, and glipizide would result in higher weight change compared to initiation and adherence to GLP‐1RAs: 4.36 kg (95% CI −0.10, 10.11), 5.26 kg (95% CI −0.12, 11.88), 4.17 kg (95% CI −0.02, 10.14), and 5.75 kg (95% CI 1.35, 11.13), respectively. Estimates of population average (absolute) weight gain at 6‐, 12‐, and 24‐month follow‐up for initiation and adherence to each of the six medications are also shown in Table 2. At 24 months, we estimated that initiation and adherence to GLP‐1RAs would result in a −5.15 kg mean weight loss (95% CI −10.60, −1.36), and the SGLT‐2 strategy would result in a −6.71 kg mean weight loss (95% CI −8.38, −4.34) (Table 2). Weight change was limited for the other medications: DPP4s had evidence for weight loss at 6 months of −0.89 kg (95% CI −1.41, −0.32) but not thereafter; glimepiride would result in mean weight gain of 0.88 kg (95% CI 0.44, 1.22) at 6 months and 1.01 kg (0.32, 1.51) at 12 months. Figure 2 displays the model‐estimated mean weight change with 95% CIs for each of the medication classes over time.

**TABLE 2:** Associations (95% CI)* of adherence to antidiabetes treatments with absolute weight change (kg) at 6, 12, and 24 months after initiation and relative weight change compared to GLP‐1RAs.

Treatment	6 months	12 months	24 months
	***β*** (95% CI)	***β*** (95% CI)	***β*** (95% CI)
Absolute weight change			
GLP‐1RA	**−2.58 (−3.66, −1.81)**	**−2.56 (−4.69, −1.03)**	**−5.15 (−10.60, −1.36)**
DPP4	**−0.89 (−1.41, −0.32)**	−0.06 (−0.77, 0.62)	−0.79 (−2.31, 0.84)
Glimepiride	**0.88 (0.44, 1.22)**	**1.01 (0.32, 1.51)**	−0.98 (−2.66, 0.89)
Glipizide	0.38 (−0.08, 0.69)	0.28 (−0.39, 0.72)	0.60 (−0.88, 1.64)
Insulin	**1.25 (0.46, 1.99)**	1.12 (−0.46, 2.49)	0.11 (−3.17, 3.76)
SGLT‐2	**−2.69 (−3.50, −1.80)**	**−3.38 (−4.54, −2.44)**	**−6.71 (−8.38, −4.34)**
Relative weight change compared to GLP‐1RAs			
GLP‐1RA	0.00 (ref)	0.00 (ref)	0.00 (ref)
DPP4	**1.69 (0.73, 2.91)**	**2.50 (0.77, 4.76)**	4.36 (−0.10, 10.11)
Glimepiride	**3.46 (2.53, 4.58)**	**3.56 (1.82, 5.76)**	4.17 (−0.02, 10.14)
Glipizide	**2.96 (1.94, 4.06)**	**2.83 (1.17, 5.00)**	**5.75 (1.35, 11.13)**
Insulin	**3.83 (2.72, 5.11)**	**3.68 (1.51, 6.27)**	5.26 (−0.12, 11.88)
SGLT‐2	−0.11 (−1.20, 1.37)	−0.82 (−2.73, 1.50)	−1.56 (−5.66, 4.21)
Relative risk of gaining ≥ 5% baseline weight change compared to GLP‐1RAs			
GLP‐1RA	1.0 (ref)	1.0 (ref)	
DPP4	1.85 (0.93, 4.03)	1.44 (0.59, 5.32)	
Glimepiride	**3.12 (1.90, 6.87)**	1.75 (0.73, 6.75)	
Glipizide	**3.24 (1.84, 6.84)**	2.17 (0.86, 8.03)	
Insulin	**4.89 (2.83, 10.35)**	**2.77 (1.16, 10.73)**	
SGLT‐2	0.65 (0.28, 1.47)	0.52 (0.13, 2.13)	

*Note*: Results in bold do not include the null, *p* < 0.05.

Abbreviations: DPP4, dipeptidyl peptidase IV inhibitors; GLP‐1RAs, glucagon‐like peptide‐1 receptor agonists; ref, reference; SGLT‐2, sodium‐glucose cotransporter 2 inhibitors.

* Estimated mean (absolute) weight change using the described IPW of repeated outcomes modeling procedure adjusting for time and baseline covariates. 95% CIs were calculated from 1000 bootstrapped samples. Results for relative risk of gaining ≥ 5% of baseline weight compared to GLP1‐RAs include data for 6 and 12 months only due to sample size decline resulting in wide CIs at the 24‐month time period.

Compared to patients prescribed GLP‐1RAs, the relative risk (RR) of gaining ≥ 5% baseline weight was significantly higher for patients prescribed glimepiride (RR 3.12; 95% CI 1.90, 6.87), glipizide (RR 3.24; 95% CI 1.84, 6.84), and long‐acting insulin (RR 4.89; 95% CI 2.83, 10.35) at 6 months. The RR of gaining ≥ 5% baseline weight was significantly higher for patients prescribed long‐acting insulin compared to GLP‐1RAs at 12 months (RR 2.77; 95% CI 1.16, 10.73).

### 3.2 Secondary Outcome Analyses

For secondary analyses targeting only effects of initiating each medication (regardless of subsequent adherence), effect estimates were somewhat attenuated compared to the primary analysis that included information about medication adherence (Table S4). However, similar to primary results, compared to GLP‐1RAs, each of the medication subclasses except SGLT‐2s was associated with greater weight gain at each of the follow‐up time points. Similarly, estimates of the population average (absolute) weight change over time showed similar results but with attenuated point estimates. For example, in these secondary analyses, the estimated mean weight loss under initiation of GLP‐1RAs was −2.39 kg (95% CI −3.17, −1.68) and under initiation of SGLT‐2s was −3.15 kg (95% CI −4.12, −2.29) at 24 months.

## 4 Discussion

In this study leveraging EHR data on patients with recently prescribed metformin, GLP‐1RAs and SGLT‐2s were associated with similar patterns of weight loss over 24 months of follow‐up. When the weight change associated with initiation of and adherence to medications was compared to GLP‐1RAs as a reference, we found that all subclasses other than SGLT‐2s were associated with higher weight change over all time periods of follow‐up, with a range of 4.36 kg for DPP4s to 5.75 kg for glipizide at 24 months of follow‐up. We estimated a mean absolute weight loss under GLP1‐RAs of 5.15 kg and a mean absolute weight loss of 6.71 kg under SGLT‐2s at 24 months. Glimepiride was associated with weight gain at 6 and 12 months of 0.88 kg and 1.01 kg but not at 24 months, while DPP4 medications were associated with weight loss at 6 months (−0.89 kg) that was not sustained at 12 and 24 months.

This study provides an important and pragmatic evaluation of the initiation of and adherence to glucose‐lowering medications over a longer follow‐up than many previous studies. In addition, by studying the use of these medications in patients recently prescribed metformin, the conclusions have direct implications for typical clinical practice. The results from the present study are consistent with prior findings [12, 28, 29]. For example, in a recent meta‐analysis of RCTs of adults with overweight or obesity and T2D, patients receiving monotherapy with either GLP‐1RAs or SGLT‐2s compared to placebo had mean weight losses of −1.47 kg to −11.47 kg and −1.66 kg to −2.44 kg, respectively, with a median duration of follow‐up across all studies of 24 weeks [28]. In another meta‐analysis of 14 RCTs of adults with T2D with follow‐up of 12 – 102 weeks, SGLT‐2s resulted in significant weight loss (weighted mean difference of −2.73 kg) [29]. There are several important differences between previous studies and this study. First, many prior studies had shorter treatment durations and reported weight change primarily between 24 and 52 weeks [12, 29], with some as short as 12 weeks [28]. Second, studies explored varied uses of GLP‐1RAs and SGLT‐2s, including (1) monotherapy or in combination with other glucose‐lowering medications, (2) comparison of these treatments to placebo‐only [12, 29], or (3) comparisons of treatment in combination with lifestyle modifications (e.g., diet, physical activity) [28]. Our study only included patients who had received a prescription for metformin in the 13 months prior to their new initiation of an additional treatment for T2D.

We estimated that both GLP‐1RAs and SGLT‐2s were associated with similar weight loss across time compared to the other subclasses examined. These differences were consistent across time with absolute differences in body weight lost for SGLT‐2s compared to GLP‐1RAs across 6, 12, and 24 months of only −0.11 kg, −0.82 kg, and −1.56 kg. This aligns with similar findings from a 2020 systematic review and meta‐analysis of RCTs that reported similar reductions in body weight between GLP1‐RA and SGLT‐2 drug classes in patients taking metformin [30]. Collectively, the findings from our study and Patoulias and colleagues [30] provide important clinical insights and suggest that patients may experience similar weight change over time with either GLP‐1RAs and SGLT‐2s when used as a treatment for T2D. Given the importance of weight loss for optimizing treatment of T2D [5], our study can help clinicians and patients evaluate potential weight effects anticipated over time when using metformin in combination with either GLP‐1RAs or SGLT‐2s. However, other important factors may also drive treatment decisions for prescribing GLP‐1RAs and SGLT‐2s including side effects (e.g., nausea and diarrhea, hypoglycemic events, mycotic infections) and other potential benefits (e.g., blood pressure control) [28, 30].

In this study, sulfonylureas and insulin were the most commonly prescribed subclasses (74% in total) in combination with metformin; DPP4s, GLP‐1RAs, and SGLT‐2s together made up 26% of all initiated prescriptions. These findings mirror recent national prescribing trends [31] and are unsurprising given that prior to 2022, clinical management of hyperglycemia in T2D was primarily focused on prevention or delay of diabetes‐related complications and not weight management [32]. Moreover, costs of newer drug classes (e.g., GLP‐1RAs, SGLT‐2s) and healthcare provider preferences have been cited as barriers to their routine use [33]. Nonetheless, diabetes and obesity are costly, with roughly $413 billion spent annually on diabetes care in the U.S. [34] and ~ $173 billion spent annually for adult obesity [35]. Given the recent emphasis on pharmacologic treatment for obesity as part of the treatment approach for T2D, our study provides important clinical insights across a 2‐year treatment period to support the effectiveness of SGLT‐2s and GLP‐1RAs for achieving significant weight loss. However, it should be noted that our study included relatively few patients who received semaglutide, a GLP‐1RA that has been associated with extensive weight loss and is FDA approved for the treatment of obesity [28]. Given that newer GLP‐1RAs such as semaglutide have been associated with significant weight loss (upwards of −11.51 kg) [28], we would expect semaglutide to have greater weight loss than SGLT‐2s. Moreover, our study also did not include tirzepatide, a newer agent that has been approved for both the treatment of diabetes and obesity and is also associated with significant weight loss [36]. Future research with larger study populations should explore individual medications within the subclasses of SGLT‐2s and GLP‐1RAs to determine precise differences in expected weight loss.

This study has several important limitations. First, as data were abstracted from EHR data, we did not have data available on medication dispensing. Medication adherence was determined by use of prescription data over time, including use of information provided in prescriptions (days' supply, number of pills provided, refills), as well as repeated prescriptions over time, allowing for a grace period between medication refills. We included a strict definition for adherence and considered patients to be nonadherent if they did not have the medication available by the end of the grace period (i.e., determined by the information available from the electronic prescription record). When prescription information was missing, it was assumed that patients had only a 1‐month supply. This assumption may have misclassified patients as nonadherent when they were in fact adherent, likely biasing our results to the null. However, it was assumed that all medication subclasses would have similar missingness; thus, biases should be similar across the subclasses compared. Second, access to routine data on medication dose was not available to explore dose response. Third, it was not required for patients to continue treatment with metformin once they initiated a new medication for diabetes treatment, and some patients may have stopped their metformin at the time of or prior to the receipt of a new medication. Fourth, not all patients had diagnostic codes documented for T2D; however, it was assumed that all patients in the analysis had T2D, considering that they were prescribed metformin and another medication commonly used to treat T2D. Nonetheless, patients may have had other indications for metformin in addition to GLP‐1RAs or SGLT‐2s (e.g., polycystic ovarian syndrome, obesity) [37]. Fifth, as with all observational study designs, there is the possibility of residual confounding, which in this study may have included a patient's desire or intention to lose weight. To address this concern, our models included multiple variables available within the EHR to address potential confounding, as well as the presence of outcome measurement. Lastly, “truncation by death” is a longstanding challenge for causal inference with outcomes like weight change even in “perfectly executed” randomized trials. Often, outcomes are not meaningfully defined for individuals who die between baseline and the selected outcome time, such that true population average causal effects are actually not defined relative to that entire population [38, 39]. In our case, “censoring by death” and excluding deaths in the analysis did not meaningfully change results.

## 5 Conclusion

An average absolute weight loss of 5.72 kg associated with initiation and adherence to GLP‐1RAs and an average weight loss of 6.51 kg associated with SGLT‐2s over 24 months could be expected for adults with T2D taking metformin. Sulfonylureas, insulin, and DPP4 medications are likely to result in higher weight over a 24‐month period compared to GLP‐1RAs. These findings highlight clinical implications for prescribing GLP‐1RAs and SGLT‐2s to support weight loss in adults with T2D.

## Author Contributions

S.L.R.‐S., J.P., J.G.Y., L.C.B., M.F.D., D.M.J., C.P., J.P.B., and W.J.H. were involved in the conception, design, and conduct of the study and the analysis and interpretation of the results. W.S.J. was involved in the analysis and interpretation of the results. N.M.S., S.L.R.‐S., J.P.B., and W.J.H. wrote the first draft of the manuscript; all authors edited, reviewed, and approved the final version of the manuscript. J.P.B. is the guarantor of this work and, as such, had full access to all the data in the study and takes responsibility for the integrity of the data and the accuracy of the data analysis.

## Conflicts of Interest

W.J.S. reports grant funding from Boehringer Ingelheim, Bayer, Merck, and Novartis outside the submitted work. The other authors declare no conflicts of interest.

## Supporting information


**Data S1:** oby70022‐sup‐0001‐supinfo.docx.

## Data Availability

The data that support the findings of this study are available on request from the corresponding author. The datasets generated during and/or analyzed during the current study are not publicly available because they were obtained from electronic health records of medical systems that only authorized the use of the data for the present analysis.
