# Supplementary material for: Comparative Weight Change With Initiation and Adherence to Common Medications for Type 2 Diabetes
Source: Obesity (Silver Spring). 2025 Oct 16;33(11):2205–13. doi: 10.1002/oby.70022 (PMC12559777; doi:10.1002/oby.70022)
Supplement: Supplementary file 1 — Data S1: oby70022‐sup‐0001‐supinfo.docx. [file OBY-33-2205-s001.docx]

**Online Supplementary Materials**

**Comparative Weight Change with Initiation and Adherence to Common Medications for Type 2 Diabetes**

| **Supplementary Table S1. Number of Patients Included in the Analysis by Health System**   \| Clinical site \| Years included^1^ \| N \| \| --- \| --- \| --- \| \| Duke University Medical Center \| 2014-2019 \| 6474 \| \| Kaiser Permanente Colorado \| 2010-2019 \| 5475 \| \| University of Florida \| 2015-2019 \| 3645 \| \| Vanderbilt University Medical Center \| 2010-2019 \| 3575 \| \| AdventHealth \| 2014-2019 \| 1513 \| \| University of Miami \| 2012-2019 \| 792 \| \| Tallahassee Memorial HealthCare \| 2012-2019 \| 644 \| \| Orlando Health System \| 2014-2019 \| 483 \|   **Supplementary Table S2.** Specifications of the target trial and its “emulation” with observational electronic health record data. | | |
| --- | --- | --- | --- | --- | --- | --- | --- | --- | --- | --- | --- | --- | --- | --- | --- | --- | --- | --- | --- | --- | --- | --- | --- | --- | --- | --- | --- | --- | --- |
| Protocol component | Target trial specification | Emulation |
| Eligibility criteria | - Age 20 – <80 years - No previous history of anti-diabetes medications other than metformin - Weight measure at baseline - No cancer diagnosis (other than non-melanoma skin cancer) 1 year prior to initiation - No pregnancy 1 year prior to initiation - No bariatric surgery 3 years prior to initiation - Indicated for monotherapy of one of the considered medications - Initiated metformin within the past 13 months - Plans to start one and only one of study-eligible medications within the month (but has not decided which one) | - Age 20 – <80 years - ≥1 encounter at least 6 months prior to first prescription - Weight measured within a 3-month period prior to initiation of second line monotherapy - No cancer diagnosis (other than non-melanoma skin cancer) 1 year prior to initiation - No pregnancy 1 year prior to initiation - No bariatric surgery 3 years prior to initiation - Initiated metformin within the past 13 months (and then initiated a new subclass of interest) - Actually initiated only one of the considered medications |
| Baseline | - Randomization would occur once all eligibility criteria are met | - Baseline is the date of treatment initiation (new subclass) once all eligibility criteria are met |
| Treatment strategies | - Initiate only 1 medication (defined by the treatment arm) and - Adhere to/continue to take the initiated medication daily, allowing for a 1-month “grace period” (treatment breaks of no more than one month duration at patient/doctor discretion) - Upon pregnancy or bariatric surgery, continued use of the medication is no longer required, can stop or continue at doctor discretion.   Medication defining each of six treatment arms: 1) any DPP4, 2) any long-acting insulin, 3) any SGLT-2, 4) Glimepiride, 5) Glipizide, or 6) any GLP1-RA (reference). | - Date of medication initiation was the date of first prescription - We estimated the amount of time a patient had medication using information on number of pills, days’ supply, and number of refills from the prescription. Patients were considered adherent during the time when they had medication on hand based on these calculations; the month following the end of their supply of medicine was the grace period. Upon pregnancy or bariatric surgery patients considered adherent to the strategy from that point forward regardless of their prescription data. |
| Treatment assignment | - Randomly assigned to a treatment strategy at baseline | - Treatment not assigned randomly (requires confounding adjustment) |
| Outcome | - Weight change compared to baseline weight measured 6-, 12-, and 24-months following study initiation. | - Same as target trial |
| Follow-up period | - Starts at baseline and ends at the end of available data, death, or 2 years after baseline, whichever comes first | - Same as target trial |
| Causal effects of interest* | - Primary (per-protocol): The effect that would be unbiasedly estimated by comparing outcome means at each time of interest across each arm (relative to the reference) had the target trial been perfectly executed (perfect adherence to the assigned treatment strategy and complete measurement of outcomes)* - Secondary (intention-to-treat): The effect that would be unbiasedly estimated by comparing outcome means at each time of interest across each arm had there been perfect adherence only to the initiation part of the assigned strategy and complete measurement of outcomes. | - Same as target trial |
| Analysis plan* | In an imperfect execution of the target trial with nonadherence to the assigned strategy and a nonmonotonic outcome missingness process, same as the “emulation” but perhaps without the need to adjust for baseline confounders. | See details in the statistical analysis section of main text. |
| ***What constitutes a meaningful notion of causal effect when deaths occur prior to the outcome of interest is a topic on ongoing debate even in the context of analyzing randomized trials (refs). When deaths do occur, and the outcome is weight gain (undefined post death) there is no causal effect we can define that is guaranteed unbiasedly estimated even in a perfect execution of the target trial. We took a common approach of censoring by death in our main analysis and excluded deaths in a sensitivity analysis. More work is needed to articulate clinically meaningful effect notions of antidiabetic medications on weight gain in populations where interim deaths may occur regardless of study design.** | | |

By the definition of our treatment strategies (Supplementary Table 1) and our rules for classifying an individual as still adherent by the prescription data as outlined above, an individual could only possibly be artificially censored in a given month (i.e. considered “nonadherent”) if 1) that month constituted the end of a one month medication grace period for a patient and they did not receive a new prescription for their initiated medication in that month and 2) the patient had no evidence up to that month of pregnancy or bariatric surgery (events that under our strategy definitions would not require continuing the medication). Thus, these “adherence models” were only fit using person-month records meeting both conditions 1) and 2). As described elsewhere, it is not generally straightforward to stabilize inverse probability weights (IPWs) in the context of dynamic time-varying treatment strategies (1), however, we did stabilize the “outcome measurement” component of the weight resulting in overall stabilized IPWs.

**References**

1. Petimar J, Young JG, Yu H, Rifas-Shiman SL, Daley MF, Heerman WJ, Janicke DM, Jones WS, Lewis KH, Lin PD, Prentice C, Merriman JW, Toh S, Block JP. Medication-Induced Weight Change Across Common Antidepressant Treatments : A Target Trial Emulation Study. Annals of Internal Medicine 2024;177:993-1003

**Supplementary Table S3. Covariates included in analyses**

| Covariate | Baseline | Follow-up | Terms^1^ |
| --- | --- | --- | --- |
| ***Demographics*** |  |  |  |
| Site | Yes | *--* | Indicator for each site |
| Year | Yes | *--* | Indicator for each year |
| Age | *Yes* | *--* | Continuous spline |
| BMI | *Yes* | *Yes* | Continuous spline |
| Sex | *Yes* | *--* | Male, Female |
| Race | *Yes* | *--* | Asian, Black, >1 race or other race, White |
| Ethnicity | *Yes* | *--* | Hispanic, Non-Hispanic |
| Medicaid | *Yes* | *Yes* | Yes, No |
| Current smoker | *Yes* | *Yes* | Yes, No |
| Number of encounters | *Yes* | *Yes* | 4-category |
| Weight change in past 6m | *Yes* | *Yes* | Loss, Same, Gain |
| ***Diagnoses^1^*** |  |  |  |
| Carlson comorbidity index >1 | *Yes* | *Yes* | Dx <12 months ago + dx ≥12 months ago + interaction |
| Type 2 diabetes | *Yes* | *Yes* | Dx <12 months ago + dx ≥12 months ago + interaction |
| Microvascular comp | *Yes* | *Yes* | Dx <12 months ago + dx ≥12 months ago + interaction |
| OT_diabetic | *Yes* | *Yes* | Dx <12 months ago + dx ≥12 months ago + interaction |
| Asthma | *Yes* | *Yes* | Dx <12 months ago + dx ≥12 months ago + interaction |
| Heart failure | *Yes* | *Yes* | Dx <12 months ago + dx ≥12 months ago + interaction |
| CAD (Coronary Artery Disease) | *Yes* | *Yes* | Dx <12 months ago + dx ≥12 months ago + interaction |
| Chronic kidney disease | *Yes* | *Yes* | Dx <12 months ago + dx ≥12 months ago + interaction |
| Hyperthyroidism | *Yes* | *Yes* | Dx <12 months ago + dx ≥12 months ago + interaction |
| Hypothyroidism | *Yes* | *Yes* | Dx <12 months ago + dx ≥12 months ago + interaction |
| Abnormal glucose | *Yes* | *Yes* | Dx <12 months ago + dx ≥12 months ago + interaction |
| Severe obesity | *Yes* | *Yes* | Dx <12 months ago + dx ≥12 months ago + interaction |
| PCOS (Polycystic Ovarian Syndrome) | *Yes* | *Yes* | Dx <12 months ago + dx ≥12 months ago + interaction |
| Cancer | *--* | *Yes* | Dx <12 months ago + dx ≥12 months ago + interaction |
| Bariatric surgery | *--* | *Yes* | Dx <12 months ago + dx ≥12 months ago + interaction |
| Pregnancy | *--* | *Yes* | Dx <12 months ago + dx ≥12 months ago + interaction |
| ***Medications*** |  |  |  |
| Antiseizure medication prescription^2^ | *Yes* | *Yes* | Rx <15 months ago |
| Antidepressant medication prescription^2^ | *Yes* | *Yes* | Rx <15 months ago |
| Antipsychotic medication prescription^2^ | *Yes* | *Yes* | Rx <15 months ago |
| Antihypertensive medication prescription^2^ | *Yes* | *Yes* | Rx <15 months ago |
| Short-acting insulin | *--* | *Yes* | Rx in current month, no rx in current month |
| Weight loss medication | *--* | *Yes* | Rx in current month, no rx in current month |
| Steroid | *--* | *Yes* | Rx in current month, no rx in current month |
| Metformin | *--* | *Yes* | Rx in current month, no rx in current month |
| Other diabetes prescription^3^ | *--* | *Yes* | Rx in current month, no rx in current month |
| Months first metformin rx to new DM subclass | *Yes* | *--* | Continuous |
| Months last metformin rx to new DM rx subclass | *Yes* | *--* | Continuous |

| 1. Diagnosis variables included terms for dx <12 months ago to adjust for recent history, dx ≥12 months ago to adjust for older history, and an interaction term to adjust for having both a recent and an older history with the condition. |
| --- |
| 2. 15 months was used because we assumed, conservatively, that each patient could be given a prescription for up to 12 months. We added an additional 3 months as a grace period (i.e., allowing each patient up to 3 months to refill the prescription). |
| 3. Using the same rules for calculating the length of time of each prescription as for the main treatment strategy. |
|  |
| *IPW models (to predict having a weight, being in a grace period) include BL and TV covariates* |
| *Marginal structural models (main results) include BL covariates and stabilized weights from IPW.* |

**Supplementary Table S4. Associations* (95% CI) of initiating-only anti-diabetes treatments with absolute and relative weight change after 6, 12, and 24 months**

| Treatment | 6 months | 12 months | 24 months |
| --- | --- | --- | --- |
|  | β (95% CI) | β (95% CI) | β (95% CI) |
| ***Absolute weight change*** |  |  |  |
| GLP-1RA | **-1.85 (-2.23,-1.50)** | **-1.85 (-2.31,-1.40)** | **-2.39 (-3.17,-1.68)** |
| DPP4 | **-0.92 (-1.16,-0.66)** | **-0.94 (-1.22,-0.65)** | **-1.66 (-2.11,-1.21)** |
| Glimepiride | **0.21 (0.02, 0.39)** | 0.09 (-0.16, 0.32) | **-0.81 (-1.15,-0.52)** |
| Glipizide | -0.01 (-0.17, 0.16) | -0.19 (-0.40, 0.02) | **-1.12 (-1.43,-0.81)** |
| Insulin | -0.03 (-0.27, 0.21) | 0.22 (-0.10, 0.53) | -0.42 (-0.89, 0.04) |
| SGLT-2 | **-2.14 (-2.52,-1.80)** | **-2.35 (-2.84,-1.89)** | **-3.15 (-4.12,-2.29)** |
| ***Relative weight change compared to GLP-1RAs*** |  |  |  |
| GLP-1RA | 0.00 (ref) | 0.00 (ref) | 0.00 (ref) |
| DPP4 | **0.94 (0.51, 1.37)** | **0.91 (0.35, 1.44)** | 0.74 (-0.11, 1.63) |
| Glimepiride | **2.06 (1.64, 2.46)** | **1.94 (1.42, 2.48)** | **1.58 (0.81, 2.40)** |
| Glipizide | **1.85 (1.44, 2.28)** | **1.66 (1.17, 2.15)** | **1.28 (0.50, 2.13)** |
| Insulin | **1.82 (1.38, 2.25)** | **2.07 (1.48, 2.63)** | **1.97 (1.17, 2.85)** |
| SGLT-2 | -0.29 (-0.84, 0.19) | -0.50 (-1.17, 0.10) | -0.76 (-1.96, 0.41) |

*Mean weight change estimated from models adjusting for time and baseline covariates. Time-varying covariates were adjusted for by applying inverse probability weights. 95% confidence intervals were calculated from 1000 bootstrapped samples
